# Structural variants shape the genomic landscape and clinical outcome of multiple myeloma

**DOI:** 10.1038/s41408-022-00673-x

**Published:** 2022-05-30

**Authors:** Cody Ashby, Eileen M. Boyle, Michael A. Bauer, Aneta Mikulasova, Christopher P. Wardell, Louis Williams, Ariel Siegel, Patrick Blaney, Marc Braunstein, David Kaminetsky, Jonathan Keats, Francesco Maura, Ola Landgren, Brian A. Walker, Faith E. Davies, Gareth J. Morgan

**Affiliations:** 1 Department of Biomedical Informatics, University of Arkansas for Medical Sciences, Little Rock, AR USA; 2 Winthrop P. Rockefeller Cancer Institute, University of Arkansas for Medical Sciences, Little Rock, AR USA; 3 Perlmutter Cancer Center, NYU Langone Health, New York, NY USA; 4 Institute of Cellular Medicine, University of Newcastle upon Tyne, Newcastle, UK; 5 Integrated Cancer Genomics Division, Translational Genomics Research Institute, Phoenix, USA; 6 Sylvester Cancer Center University of Miami, Miami, FL USA; 7 Division of Hematology Oncology Indiana University, Indianapolis, IN USA

**Keywords:** Genetics research, Cancer genetics

## Abstract

Deciphering genomic architecture is key to identifying novel disease drivers and understanding the mechanisms underlying myeloma initiation and progression. In this work, using the CoMMpass dataset, we show that structural variants (SV) occur in a nonrandom fashion throughout the genome with an increased frequency in the t(4;14), *RB1*, or *TP53* mutated cases and reduced frequency in t(11;14) cases. By mapping sites of chromosomal rearrangements to topologically associated domains and identifying significantly upregulated genes by RNAseq we identify both predicted and novel putative driver genes. These data highlight the heterogeneity of transcriptional dysregulation occurring as a consequence of both the canonical and novel structural variants. Further, it shows that the complex rearrangements chromoplexy, chromothripsis and templated insertions are common in MM with each variant having its own distinct frequency and impact on clinical outcome. Chromothripsis is associated with a significant independent negative impact on clinical outcome in newly diagnosed cases consistent with its use alongside other clinical and genetic risk factors to identify prognosis.

## Introduction

The transcriptional abnormalities giving rise to inter-patient variability in the outcome of multiple myeloma (MM) are driven by acquired genetic hits. Many of these events have been identified using next generation sequencing, which has successfully defined relevant single-nucleotide variants, indels and copy number abnormalities in newly diagnosed myeloma (NDMM) [1, 2]. However, much less is known about the role of structural events, which mostly occur in the non-coding regions and are best characterised by whole genome sequencing.

Deciphering the genomic architecture of myeloma is key to identifying novel disease drivers and to gain insights into the mechanisms underlying myeloma initiation and progression. Structural variants (SV) are generally defined as regions of DNA, approximately 1 kb or larger, that can include inversions, balanced translocations or genomic imbalances (insertions and deletions), and can be divided into simple and complex events depending on the number of breakpoints or chromosomes involved. A significant body of evidence points towards the importance of chromosomal rearrangements in MM but their full consequences on chromatin structure, topologically associated domains and transcriptional networks have yet to be fully explored.

Until now the best characterized of the structural events in MM are the primary translocations to the immunoglobulin gene (Ig) loci, which lead to oncogene overexpression by hijacking the super-enhancer sites located at these loci. These recurrent structural variants place the Ig super-enhancers in proximity to one of five oncogenes generating the t(4;14)[*NSD2 FGFR3/IGH*](15%), t(11;14)[*CCND1/IGH*](20%), t(14;16)[IGH/*MAF*](5%), t(6;14)[*CCND3/IGH*], and t(14;20)[IGH/*MAFB*] [1]. Secondary SVs are also thought to play a key role in MM disease progression and the best characterized of these occur at 8q24, the site of *MYC*.

In other non-hematologic cancers, SVs have been noted to be complex and to involve more than two sites. These complex events have also been seen in MM but their clinical significance has been uncertain [3]. Molecular characterisation of such complex structural events has identified focal copy number changes at the breaks, which can involve either gain or loss of copy number. These complex SVs have been termed chromoplexy when there is associated copy number loss and templated insertion when there is copy number gain [4]. A further complex event chromothripsis (“chromosome shattering”) is defined by localized clustered rearrangements associated with loss of heterozygosity and copy number oscillation [5]. In a recent report mapping SVs in a whole genome dataset of MM, we showed that the incidence of chromothripsis, complex templated insertion (affecting > 2 chromosomes), chromoplexy, is approximatively 24, 19, and 10% [6]. Both chromothripsis and templated insertion may affect expression levels but much remains to be understood about the full contribution of structural events to the patterns of gene deregulation and clinical outcome of MM.

Gene expression within plasma cells, as in all specialized cells, is controlled by a set of super-enhancers, the activity of which is constrained by the three-dimensional structure of the nucleus. Under normal conditions, gene expression is constrained by both nuclear structure and localised chromatin organisation in the form of topologically associated domains (TADs) [7]. These genomic features are formed from CTCF binding sites and are important in mediating local interactions regulating gene expression. Disrupting normal TAD boundaries by cancer acquired structural events, with or without copy number change, impacts gene expression as can acquired changes in histone methylation such as H3K36 and H3K27 as a consequence of NSD2 upregulation by a t(4;14) [8]. The availability of chromatin immunoprecipitation (CHiP) sequencing data has been used to identify myeloma specific super-enhancers and High Chromosome Contact (Hi-C) sequencing provide an opportunity to determine TAD structures and understand how acquired simple and complex structural events impact gene expression and clinical outcome. In this analysis, we have taken account of TAD structures and plasma cell specific super-enhancers [9], to investigate the role of transcriptional deregulation by structural variants.

## Methods

### Sequencing analyses

#### Long-insert whole-genome sequencing (LIWGS)

Whole genome sequencing dta from 812 CoMMpass samples was aligned to hg19 using BWA (v. 0.7.17) [10] and deduplicated using samblaster (v. 0.1.24) [11]. Copy number was called with Control-FREEC (v. 11.4) [12] and structural events with Manta (v. 1.4.0) [13]. Complex structural events were called as previously described on 752 patients [6]. Telomere length was estimated in both tumors and in the germline using Telomerecat with standard settings [14].

#### Exome sequencing

Exome data for 659 samples corresponding to a subset of the CoMMpass LIWGS cases were used, as described previously [15].

#### RNA sequencing

A subset of 643 samples from the CoMMpass study had RNA sequencing available [16]. These samples where aligned to hg38 using STAR (2.5.1b) [17] and quality controlled using QoRTS (v1.2.42) [18] with alignment and quantification of gene read count with Salmon (v0.7.2) [19]. Normalization of counts and differential gene expression analysis was performed using DESeq2 (v1.14.1) [20]. Gene set enrichment analysis was performed using the R package fgsea (v1.6.0) [21].

### TAD identification

Hi-C data was obtained for the U266 and RPMI-8226 cell line from Wu et.al and was processed as published. To identify disruption of TAD boundaries and associated gene upregulation we used calls from Manta and assigned them to within a TAD structure as defined by the Hi-C data, from published cell line data. We identified neo-TAD formation and compared expression of genes within the TADs depending on the presence of a rearrangement containing a gene that was significantly upregulated compared to all other genes using an ANOVA test with FDR adjusted *p* values (*p* < 0.05) [22].

### Statistical analysis

The distribution of variants between different subtypes of MM was evaluated, using Fisher’s exact test or Kruskal-wallis test. Stepwise Cox regression in both directions, based on Akaike information criterion (AIC), using classical risk factors estimated the effects of significant covariates for time-to-event outcome. The final Cox model consisted only of statistically significant factors at a level of *p* < 0.05. An additional bootstrap was performed using the rms package (B = 100) and corrected indices (Dxy and r^2^) computed. Correlation between mutated genes, cytogenetic abnormalities, complex rearrangements, and clinical features using Bayesian inference was determined using the program “JAGS” and the R-interface Bayesmed. The probability of the observed data under the null hypothesis versus the alternative hypothesis or Bayes factor (BF) was computed. BF > 1 was considered significant. BF 1–3, 3–20, 20–150, and > 150 were considered weak, positive, strong and very strong associations respectively. Further details may be found in the Supplemental material.

## Results

### Simple and complex structural variations are common features of the NDMM genome and have distinct molecular associations

**Fig. 1:**
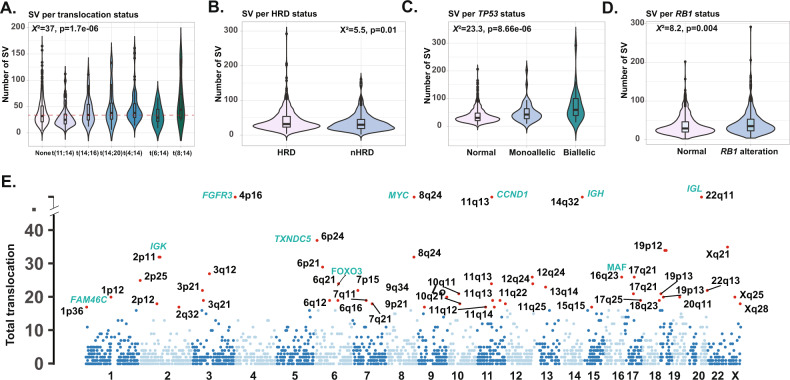
Distribution of SV across the genetic subgroups of NDMM (*n* = 812). **A** Violin plot suggesting t(4;14) have more SVs and t(11;14) fewer SVs. **B** Violin plot suggesting HRD samples have fewer SVs than nHRD samples. **C** Violin plot suggesting there are more SV in both monoallelic and biallelic *TP53* inactivated cases. **D** Violin plot suggesting SVs are associated with *RB1* alterations. **E** Plot displaying the number of translocations according to their chromosomal location highlighting hotspots of interest (genes in green).

Using the CoMMpass LIWGS dataset (*n* = 812), Supplemental Table 1, we determined the sites of structural breaks using Manta [23]. We show that the median number of SVs events per case was 31 (range 2–327) with a median of 25 intrachromosomal (0–323) and inter-chromosomal 6 (0–166). There were more structural events in cases with a t(4;14) than in cases with t(11;14) (*χ*^*2*^ = 38, *p* = 5.987e-10) and in cases that cases that lacked canonical translocations (*χ*^*2*^ = 10, *p* = 0.001), Fig. 1A.

The median number of SV across the canonical translocations groups was 38 (IQR 14–84) for t(4;14), 25 (2–63) for t(11;14), 38 (23–91) for t(14;16), and 38 (6–93) for t(14;20) [24]. Hyperdiploid (HRD) samples (Kruskal wallis test, *χ*^*2*^ = 5.5, *p* = 0.01) had fewer breakpoints than non-HRD samples, Fig. 1B. Samples with both mono and bi-allelically inactivated *TP53* had more SV than the wild type (20 (2–87) versus 27 (9–94) and 38 (14–161), *χ*^*2*^ = 23, *p* = 8.66e-06), Fig. 1C; samples with *RB1* inactivation had more mutations than those that did not (29 (2–85) vs. (35 (4–98), *χ*^*2*^ = 8.2, *p* = 0.004), Fig. 1D.

We found a negative correlation between tumor telomere lengths (TTL), a marker of DNA instability, and the number of SV and a small positive correlation with leucocyte telomere length (LTL) that is of uncertain significance, Supplemental Figs. 1–2. We did not identify a correlation between the number of SV and the exomic mutational rate but did identify a positive correlation between the number of SV and the proportion of the APOBEC mutational signature, Supplemental Fig. 3. These data suggest a background of DNA instability and DNA repair deficiency contribute to the extent of SV’s.

A group of 10% (*n* = 84) of cases were identified as having an excess of structural events, using an elbow test with a median of 102 (78–292) versus 30 (2–76) breakpoints per sample, Supplemental Fig. 4. This group correlated with *TP53* mutations (cor = 0.13, BF = 6.2), del(1q) (cor = 0.14, BF = 15), del(12p) (corr = 0.15, BF = 71), and del(17p) (corr = 0.17 BF = 396), Supplemental Fig. 5. Interestingly, 37% of these patients had neither *TP53* inactivation, *RB1* inactivation, or short TTL ( < 4.100kB) suggesting the full range of biological variants contributing to structural complexity remain to be fully defined, Supplemental Fig. 6A. Only one of these patients had an *ATM* mutation.

Complex rearrangements were evaluated in 752 cases. Chromothripsis was seen in 33%, of t(4;14), and 12% of t(11;14); chromoplexy in 19% of t(4;14), and 5% of t(11;14), respectively and templated insertion in 19% of t(4;14) and 36% and t(11;14).

The prevalence of chromothripsis was higher among patients with a *TP53* mutation (*r* = 0.22, BF = 12692), del(17p) (*r* = 0.21, BF = 91754), del(12p) (*r* = 0.17, BF = 575), del(1p) (*r* = 0.14, BF = 25), del(6q) (*r* = 0.12, BF = 2.3), del(16q) (*r* = 0.11, BF = 1.7), del(13q) (*r* = 0.1, BF = 1.15). The prevalence of chromothripsis was lower in patients with t(11;14) (r = −0.14, BF = 25), *KRAS* and *NRAS* mutations (*r* = −0.11 and −0.15 and BF = 2.6 and 63, respectively). 35% of patients with chromothripsis did not have a detectable mutation in *RB1*, *TP53* or have short telomeres, Supplemental Fig. 6B.

The prevalence of chromoplexy was higher among patients with a *FGFR3* mutation (*r* = 0.12, BF = 4), *DSCAML1* mutation (*r* = 0.15, BF = 33), *HYDIN* mutation (*r* = 0.15, BF = 66), del(17p) (*r* = 0.12, BF = 4.2), del(13p) (*r* = 0.13, BF = 6.9), del(6q) (*r* = 0.16, BF = 99), del(8p) (*r* = 0.13, BF = 6.9), del(10p) (*r* = 0.14, BF = 18), and t(4;14) (*r* = 0.11, BF = 11). 23% of chromoplexy patients, did not have detectable events at *RB1*, *TP53* inactivation or short telomere length, Supplemental Fig. 6C.

The prevalence of templated-insertion was higher among patients with HRD (*r* = 0.13, BF = 10), and marginally lower in patients with *DMD* mutations (*r* = −0.11, BF = 1.1), *HMCN1* mutations (*r* = −0.11, BF = 1.8), t(4;14) (*r* = −0.15, BF = 37), del(13q) (*r* = −0.15, BF = 39), del(14q23) (*r* = −0.15, BF = −28), and del(1p) (*r* = −0.11 BF = 1.5), Supplemental Fig. 5. 48% of templated-insertion cases, did not have detectable events at *RB1*, *TP53* inactivation or short telomere length, Supplemental Fig. 6D.

Combined these data suggest that SV are not evenly distributed among MM patients with t(11;14) cases having relatively bland genomes with fewer SVs. Complex SVs, t(4;14), *TP53* and *RB1* altered cases are associated with multiple SV and complex rearrangements. A significant proportion of cases had no evidence of DNA repair pathway mutations consistent with the existence of other potentially important pathways yet to be associated with these events. Templated insertions were not associated with these markers of DNA instability suggesting they are generated via a different mechanism.

### Canonical translocation breakpoints are clustered and deregulate multiple genes

We show that the sites of recurrent translocation breakpoints are not evenly distributed throughout the genome. Hotspots identified include 14q32 (*IGH*), 2p (*IGK*) and 22p (*IGL*) together with canonical rearrangements to chromosome 4p16 (*NSD2/FGFR3*), and 11q13 (*CCND1*). In addition, we found recurrent rearrangement to four additional sites (*FAM46C, TXNDC5, FOXO3, MYC)* that contained super-enhancers and provide alternatives for gene deregulation, as a consequence of chromosomal rearrangement, in addition to the super-enhancers of the Ig loci. These sites include previously described sites and additional novel sites of interest.

On chromosome 1, a chromosome recurrently associated with the adverse outcome we identified *SERTAD2* at 1p14, *TENT5C* at 1p36, *MCL1* at 1q21.3 and *NTRK1* at 1q21.1. On chromosome 6 there were clusters of SV at 6p2.1, 6p24, 6q21, and 6q24 with the sites of *TXNDC5* and *FOXO3* being the most frequently affected. On chromosome 15q21 the site of *B2M* and 15q24 the site of *ULK3* we also noted to recurrent SV. On chromosome 19, the two sites were found at 19p13, the site of *KLF2* and 19p12, the site of *ZNF675*. These novel recurrent sites occurred at low frequency (<3% of cases) and share the common feature of all containing genes that have important functions in plasma cells and previously described plasma cell super-enhancers, Fig. 1D.

### Complex SV’s occur at early disease stages but display some changes overtime

To gain insights into the pathogenic role of complex rearrangements in mediating disease progression we studied 38 paired samples obtained from presentation and relapse derived from the CoMMpass data set. We show using a paired t-test suggested that relapsed samples have more SV than at diagnosis, Supplemental Fig. 7. Thus suggesting that the frequency of SV increases between diagnosis and relapse.

**Fig. 2:**
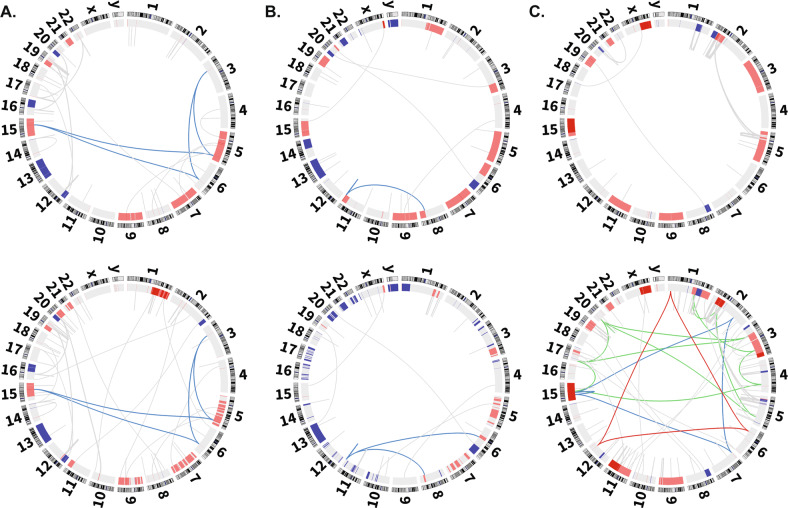
Complex SV comparison between Presentation and Relapse A. Gain of complex SV. **A** t(8;11) at presentation becomes a t(6;8;11) at relapse. **B** Stable complex SV. A t(3;5;6;15) at presentation is also detected at relapse. **C** Gain of multiple complex SVs. A relatively simple presentation sample gains multiple complex SVs at relapse including a t(2;7;15), t(1;6;12), and t(1;2;3;4;5;15;17;20).

When looking at complex events, they were detected in 16 paired cases at presentation and relapse (42%); in eleven they were detected at both time points and in five they were acquired at relapse, Fig. 2. In one of these cases, when the raw data was analysed, we found evidence for low levels the clone in the presenting sample, suggesting it was actively selected for consistent with a role as a driver. The acquisition of a complex events can play a role in relapse as illustrated by a case where relapse was associated with a significant excess of novel structural events, Supplemental Fig. 8. We did not find examples of cases where the structure of SV events changed dramatically at relapse consistent with them being relatively stable once formed.

These findings suggest that complex chained rearrangements occur throughout the natural history of MM but are predominantly present at diagnosis and are relatively early and stable molecular events. The observation that they are selected for (increase in clonal fraction) and gain additional breakpoints suggest they provide a selective advantage.

### Structural variants affecting TAD boundaries are associated with gene deregulation

**Fig. 3:**
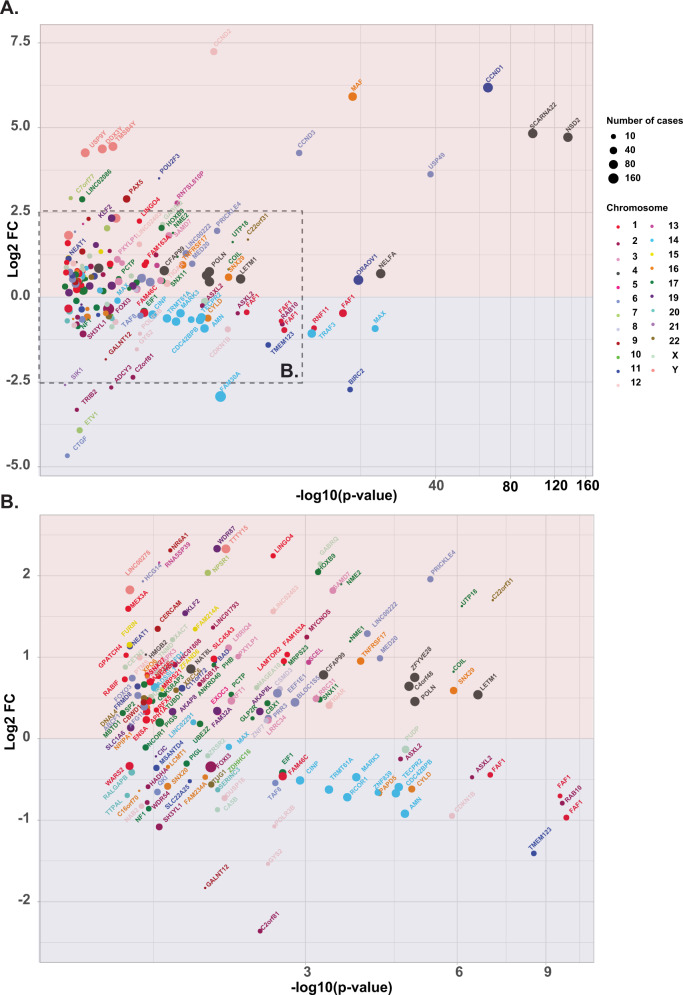
Expression changes and between cases with TAD-TAD rearrangements defined in RPMI cell lines and those that have none (*n* = 752). The x-axis represents the inverse log10 *p* value using a log-ed scale and the y axis the log2 fold change, **A** Overall **B** Focus on the central region. Genes are colored by chromosomes and points are proportional to number of cases.

To identify the range of genes recurrently dysregulated by super-enhancers, we identified all TADs using the U266 and RPMI-8226 cell lines and mapped the breakpoints previously defined by Manta. We then compared the expression of genes within the TADs between patients that had evidence of TAD-TAD rearrangement and those that did not. Changes were considered significant if they occurred in five or more cases, had a median expression >2 in at least one group, and had an FDR corrected ANOVA test with *p* < 0.05, Fig. 3-Supplemental Fig. 9.

Looking at the RPMI-8266 defined we identified canonical translocation partners (*CCND1, NSD2, MAF*) and genes that had previously been described as important in B-cell biology, such as *CCND3, CCND2*, and *PAX5*, Fig. 3. In addition to the deregulated genes routinely associated with the canonical translocations, we identified additional deregulated genes contained within rearranged TADs. The best example of this is in the t(4;14) cases where we show an upregulation of local gene expression including *LETM1*, *NELFA*, *C4orf48*, *NAT8L*, *POLN*, or *ZFYVE28* in all cases (*n* = 75), Supplemental Fig. 10. We also see a set of downregulated genes, corresponding to regions of copy number loss, mainly located on 14q including *TRAF3* and *MAX*, previously described as a tumor suppressor, 11q including *BIRC2*, and *TMEN123*, and 1p including both *FAF1* and *RNF11*. Combined, these data suggest that translocations frequently deregulate more than a single oncogene, Supplemental spreadsheet.

Looking at the U266-defined TAD, we identified significantly over-expressed genes including the canonical Ig translocation partners *FGFR3*, *CCND1*, *MAF*, and *NSD2*. The analysis also identified genes predicted by the known biology of MM including *CCND2*, *PAX5*, and *CCND3*. Other overexpressed genes identified include *SCRNA23* (within the *NSD2* locus), *NTRK1, HMGB, SAMD7, NEAT1, NAV1, MYCNOS*, UTP18, *LAG3*, *NME1*, *NME2*, *PTMS*, *UHRF1*, *GPR162*, *CERCAM*, *ZNF185*, *KCNK7*, and *FAM214A*. We also see a set of downregulated genes, corresponding to regions of copy number loss, mainly located on chromosome 14q including *TRAF3*, previously described as a tumor suppressor, 11q, including *BIRC2, TMEN123*, 13q (*RB1*), and 16q (*NUBP2*) and 9q (GALNT12), Supplemental Fig. 9.

*MAP3K14* (*NIK*) is recurrently impacted by translocations in this data set allowing us to determine the impact of the breakpoint site in relationship to TAD structure. We mapped the sites of breakpoints in relationship to the TAD boundary and expression level and demonstrate a significant difference in expression change based on translocation into or outside of the TAD boundary, Supplemental Fig. 11. This result supports the notion that not all translocations in the vicinity of a gene are pathologically relevant and those breaking a TAD boundary are more likely to deregulate expression

### The impact on outcome of complex structural events

We determined the impact of complex rearrangements on the survival of patients in the complete dataset (*n* = 752) after a median follow-up of 5.39 (5.07–5.45). The survival results show that only chromothripsis was associated with an adverse outcome in both progression-free survival (PFS, HR 1.49 (1.13–1.95), *p* = 0.004) and Overall Survival (OS, HR 1.79 (1.3–2.3), *p* = 5.68e^−5^). Patient with chromothripsis involving more than three chromosomes did significantly worse in terms of overall survival (HR 2 (1.2–3.3), *p* = 0.007) than those involving fewer chromosomes.

**Fig. 4:**
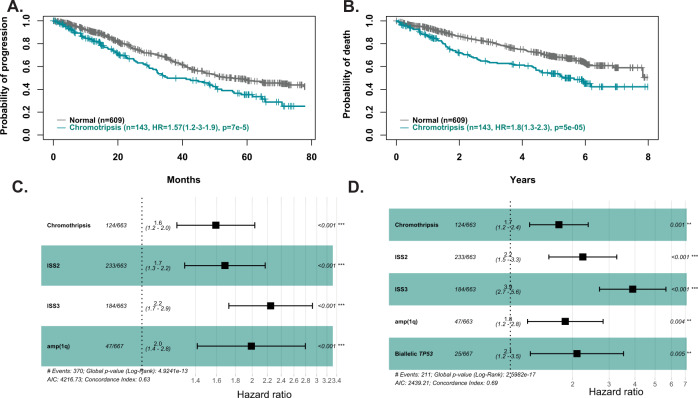
Impact of chromothripsis on outcome. **A** PFS, **B** OS, **C** forest plot representing the independent significant variables for PFS. **D** forest plot representing the independent significant variables for OS.

To look at the interaction between events we determined the impact of complex SVs on prognosis in 595 patients with a full clinical data set in the CoMMpass data and an updated follow-up. We identified chromothripsis in 23% (*n* = 137), chromoplexy in 10% (*n* = 58), and templated insertion in 37% (*n* = 219). In a multivariate analysis including chromothripsis and classical MM risk factors, such as bialleleic *TP53* inactivation, t(4;14), amp(1q) and ISS we show that the presence of chromothripsis retains its independent impact on prognosis alongside amp(1q), biallelic *TP53* inactivation, and ISS3 for OS and amp(1q) and ISS3 for PFS, Fig. 4.

Interestingly, the group carrying a high structural load was associated with an adverse outcome with a median PFS of 21.5 months (CI 18.3–29.9) compared to a median of 39.3 months (CI 35.9–43.3 *p* = 0.0001) and a 5 year-OS of 49% (CI 37–65%) compared to a median 66% (63–70% *p* = 5e-4), Supplementary Fig. 12.

### Molecular associations with chromothripsis

To identify biological features that may be associated with complex rearrangements we carried out a gene set enrichment analysis (GSEA) by comparing the expression of patients with chromoplexy, chromothripsis, and template insertion.

Across each of these groups, there was an enrichment for Oxidative phosphorylation, G2M and E2F cell cycle targets consistent with a proliferative phenotype. Other pathways consistently upregulated include *MYC* targets and *MTORC1* signalling. We also noted a downregulation of inflammatory response and proinflammatory cytokines such as TNF-ά signalling and IL6 across three groups.

**Fig. 5:**
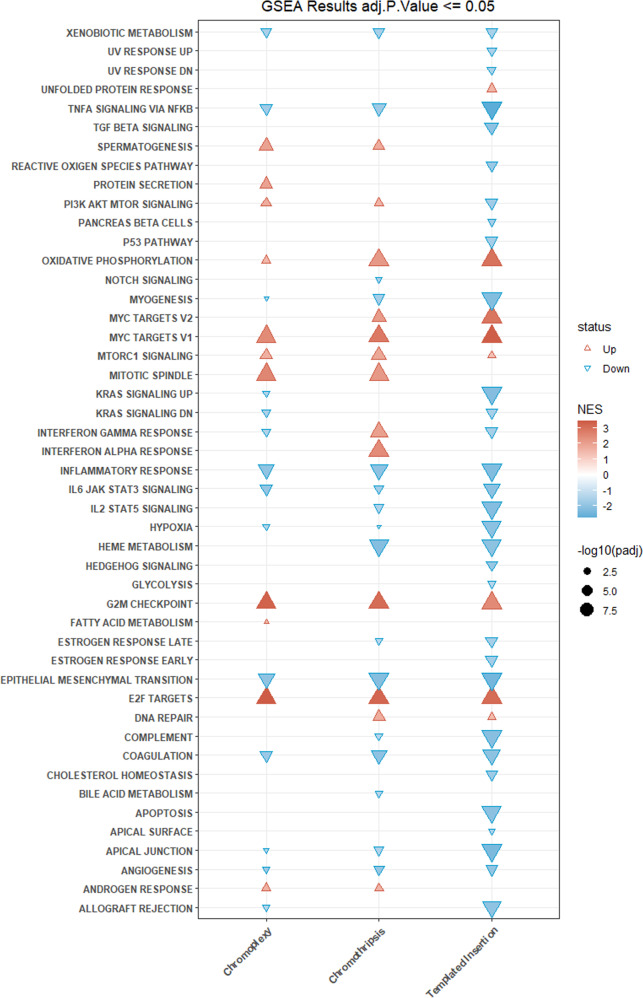
Result of the Gene Set Enrichment Analysis comparing chromothripsis, chromoplexy and templated insertion. GSEA analysis suggest some pathway are predominantly upregulated (red) or downregulated (blue) in some subsets.

When considering template insertion, apoptosis, glycolysis, KRAS signalling and the P53 pathway were downregulated. The later were particularly interesting as there TI are not associated with TP53 inactivation. On the other hand, *MYC* targets and UPR pathways were significantly upregulated in comparison to the other groups. INF-ά and γ were increased in patients with chromothripsis. Other increased pathway include Pi3K and MTOR signalling. Finally, DNA repair was upregulated in chromothripsis and templated insertion but not the chromoplexy group, Fig. 5. Combined, these data show that the three groups are differentiated by their expression signature. Although they are all associated with highly proliferative signatures, chromothripsis seems to be associated with a more inflammatory phenotype.

To address these differences further, we performed a network analysis and identified patterns of deregulation and detected co-expression of *NSD2* and *HDAC1, CCND1* and *RB1, MAF* and *MYB*, Supplemental Table 2. These data show that beyond structural events an underlying functional effect in gene expression patterns may contribute to their aetiology and outcome.

## Discussion

The analysis paints a picture of the MM genome that is substantially impacted by structural variants (SVs) that are frequent and contribute significantly to the interpatient variability in biology and clinical outcome. The distribution of SVs throughout the genome is non-random predominantly involving the Ig loci, the strong enhancers of which upregulate a series of canonical oncogenes directly attributed to the etiology of MM. In addition to the Ig loci we identified a limited number of recurrent loci that appear to also act as donors of super-enhancers deregulating genes at alternative receptor sites throughout the genome. Using TAD/TAD rearrangements, we detected both predicted and novel genes deregulated by SVs. Using MAP3K17 we show that SVs that break TAD boundaries have a greater impact on gene deregulation. Further we show that more than one gene may be impacted by the same apparent translocation based on subtle differences in the site of the breakpoints further contributing to the complexity of gene deregulation in MM and variation in clinical outcomes.

We show that complex structural events are frequent in MM with the most common being templated insertions involving amplification of regional sequences and gene overexpression. The least frequent subtype of complex rearrangements, chromoplexy, involves deletion of sequence and loss of gene expression does not seem to impact the outcome in this dataset. Chromothripsis involves focal regions of gain or loss and has a significant impact on clinical behavior and could potentially contribute to clinical risk scores.

The current prognostic score in MM is based on clinical and biochemical features and uses the serum beta-2-microglobulin value and albumin level, known as the international staging system (ISS) [25]. This prognostic score has been enhanced by incorporating cytogenetic features including del(17p), (R-ISS) and by del(17p) and gain(1q) (R2-ISS)[D'Agostino M, Cairns DA, Lahuerta JJ, Wester R, Bertsch U, Waage A, Zamagni E, Mateos MV, Dall'Olio D, van de Donk NWCJ, Jackson G, Rocchi S, Salwender H, Bladé Creixenti J, van der Holt B, Castellani G, Bonello F, Capra A, Mai EK, Dürig J, Gay F, Zweegman S, Cavo M, Kaiser MF, Goldschmidt H, Hernández Rivas JM, Larocca A, Cook G, San-Miguel JF, Boccadoro M, Sonneveld P. Second Revision of the International Staging System (R2-ISS) for Overall Survival in Multiple Myeloma: A European Myeloma Network (EMN) Report Within the HARMONY Project. J Clin Oncol. 2022 May 23:JCO2102614. doi: 10.1200/JCO.21.02614. Epub ahead of print. PMID: 35605179]. More recently mutational features have been introduced in the “Double-Hit” group that takes account of bi-allelic inactivation of *TP53* [26]. Improving further on such prognostic scores has been difficult because of the lack of recurrent variables that are sufficiently penetrant to impact clinical risk. The integration of chromothripsis into the ISS has the potential to address this deficiency because not only does it have a strong independent impact on patient’s prognosis, but it is also prevalent enough to contribute significantly to clinical risk. Further, we have shown that it may be feasible to detect chromothripsis in clinical samples using mutational signatures of copy number profiles [27]. The adverse prognostic impact of chromothripsis we describe in MM is consistent with results from other cancers where it has a strong negative impact on outcome.

There is significant inter-patient variability in the distribution of chromothripsis in MM with some cases having relatively focal events against the backdrop of an otherwise quiet genome, whereas in others chromothriptic breakpoints may co-occur with other complex events in the background of a highly rearranged genome. The impact of this type of variation needs to be explored further in future analyses.

The mechanistic basis for the adverse impact of chromothripsis on prognosis is uncertain. The molecular features of the events almost certainly significantly impact TAD structure and gene expression patterns with oscillations in copy number massive loss of chromosome fragments impacting distinct regions of the chromosomes. The net result of these features is both the activation oncogenes and inactivation of tumor suppressor genes [6]. Consistent with this hypothesis we have shown that chromothripsis is responsible for the simultaneous alterations of multiple genes.

While the time at which structural variants develop remains elusive current evidence would suggest that they are primary events in most cases, however, in some instances, additional breakpoints and structural complexity may be acquired overtime. The full relationship of structural variation to acquired DNA repair deficiency (*TP53, RB1*, and telomere attrition) is yet to be fully explored but the relationship is likely to be important.

Beyond structural variants, other mechanisms of gene dysregulation could be operating to mediate changes in gene expression. The most relevant changes we identified are mediated via epigenetic alterations such as those influencing TAD architecture leading to altered expression, but these are largely unexplored as yet in MM [28–30] Understanding the role played by such epigenetic change is likely to add important new understandings into MM biology.

The other important feature of chromothripsis the genetic background on which it develops. We show strong associations with *RB1, TP53* and telomere attrition suggesting the importance of DNA repair mechanisms. Using gene set enrichment analysis we show that cases carrying these complex rearrangements have associations with distinct expression patterns consistent with them occurring in the context and leading to distinct cellular phenotype. Gene set enrichment analyses of the association of chromothripsis, chromoplexy and templated insertions shows both distinct and common features between the different lesions. Across each of these groups, there was an enrichment for G2M and E2F cell cycle targets consistent with a proliferative phenotype and some specific evidence for deregulated pathway in some subgroups only.

In this work, we show that structural events contribute significantly to the pathogenesis and variability in the clinical outcome of MM. In particular, chromothripsis is an important molecular variable that is associated with adverse clinical outcome. The ability to detect chromothripsis in clinical samples would allow us to substantial enhance clinical risk scores and more reliably predict prognosis. Using the presence of chromothripsis as a tool to identify high-risk cases suitable for further investigation may allow us to gain further insights into the biology driving the adverse outcome for potential therapeutic targeting.

## Supplementary information

Supplemental

Supplemental spreadsheet
