# Supplementary material for: Structural variants shape the genomic landscape and clinical outcome of multiple myeloma
Source: Blood Cancer J. 2022 May 30;12(5):85. doi: 10.1038/s41408-022-00673-x (PMC9151656; doi:10.1038/s41408-022-00673-x)
Supplement: Supplementary file 1 — Supplemental [file 41408_2022_673_MOESM1_ESM.docx]

**Complex Structural Variants Shape the Genomic Landscape and Clinical Outcome of Multiple Myeloma: Supplemental data**

Cody Ashby^1,2*^, Eileen M Boyle^3*^, Michael A Bauer^1,2*^, Aneta Mikulasova^4^, Christopher P Wardell^1,2^, Louis Williams^3^, Ariel Siegel^3^, Patrick Blaney^3^, Mark Braunstein^3^, David Kaminetsky^3^, Jonathan Keats^5^, Francesco Maura^6^, Ola Landgren^6^, Brian A Walker^7^, Faith E Davies^3^ and Gareth J Morgan^3^.

^1^ Department of Biomedical Informatics, University of Arkansas for Medical Sciences, Little Rock, AR, USA

^2^ Winthrop P. Rockefeller Cancer Institute, University of Arkansas for Medical Sciences, Little Rock, AR, USA

^3^ Perlmutter Cancer Center, NYU Langone Health, New York, NY, USA.

^4^ Institute of Cellular Medicine, University of Newcastle upon Tyne, Newcastle, UK.

^5^ Integrated Cancer Genomics Division, Translational Genomics Research Institute, Phoenix, USA.

^6^ Sylvester Cancer Center University of Miami, NY, USA

^7^ Division of Hematology Oncology Indiana University, Indianapolis, IN, USA.

* Contributed equally

**Running title:** Complex structural variants and outcome in multiple myeloma.

**Corresponding Author:**

Morgan, Gareth J. Director Myeloma Research,

Morgan Lab, MSB 4.

NYU Langone Medical Center

522 1^st^ Avenue

Manhattan, New York City, NY 10016

[Gareth.morgan@nyulangone.org](mailto:Gareth.morgan@nyulangone.org)

Eileen M Boyle,

Morgan Lab, MSB 4.

NYU Langone Medical Center

522 1^st^ Avenue

Manhattan, New York City, NY 10016

Eileen.Boyle@nyulangone.org

**Supplementary Methods**

***Calling structural events***

We used Manta to call structural events in paired analysis mode using a tumor/normal pair, where it uses split and/or paired aligned reads to call an event statistically. We examined the occurrence of structural events by merging all structural calls for the CoMMpass data set and generating network maps using the igraph library in R. These results were similar in frequency to previously reported results.

**Relating breakpoint site to gene expression**

Despite the closest gene frequently being deregulated by a breakpoint, it is not simply sufficient to identify the closest gene as the “target”. Presumably, a breakpoint within a TAD could cause deregulation of any gene within that TAD. Therefore, the identification of the genes within the TAD containing a breakpoint was a high priority as we wished to determine biologically relevant gene deregulation by acquired structural events. In a similar fashion, it was of interest to know whether a given TAD contains a super-enhancer to understand its mechanistic role in gene deregulation. We used the TAD region files and annotated them with the counts of enhancers/super-enhancers using bedtools. If a TAD did not contain an enhancer or super-enhancer it was assigned the value 0, if it contained an enhancer it was assigned the number of enhancers and if it contained super-enhancers it was assigned the number of super-enhancers. The resulting file was then cross-referenced to a sample’s structural event subgraph table (generated as described above) in order to identify cases with structural events involving super-enhancers.

***NMF***

Mutational signatures were called using non-negative matrix factorization (NMF)

with counts per sample calculated for the six possible SNV types and the 16 possible 2-base sequence contexts, creating a table with 1,273 rows and 96 columns. The R package “NMF” was used for all calculations. The number of signatures was determined by running 50 iterations of the algorithm for 2-10 signatures. The number of signatures was chosen that maximized the cophenetic distance and dispersion values. One thousand (1,000) iterations of the algorithm were run for that number of signatures. Cosine similarity was used to determine the Sanger signatures that were closest to the detected signatures.

**Supplementary Results**

# **Supplementary Table 1. Baseline characteristics of 812 patients in the CoMMpass study.**

|  | Characteristic (n=812) |
| --- | --- |
| Sex ratio F:M | 1: 1.5 |
| Ethnicity | Asian: 1% (n=14)  African American: 15% (n=119)  White: 67% (n=566)  Other or unknown: 17% (n=114) |
| Median age at diagnosis | 63 (range: 27-93) |
| ISS | I 35% (n=288)  II: 35% (n=287)  III: 26% (n=210) |
| Hyperdiploidy | 40% (n=255) |
| Translocations | t(11;14): 19% (n=137)  t(14;16): 4% (n=28)  t(14;20): 1.5% (n=11)  t(4;14): 12% (n=87)  t(6;14): 1% (n=9)  t(8;14): 1% (n=6) |

**Table 2: Network analysis of gene expression data comparing a setinter-connections of genes between the groups highlighting the typical translocation partners alongside snoRNA and lncRNA the significance of which is unclear**

| gene_symbol | median_exp_event | median_exp_noevent | p | F_score | number_of_samples | padj | Delta |  |
| --- | --- | --- | --- | --- | --- | --- | --- | --- |
| ***CCND1*** | **17.55754** | **11.49996** | **9.78E-50** | **262.485** | **73** | **5.44E-47** | **6.057581** |  |
| ***NSD2*** | **16.68327** | **11.80669** | **2.79E-70** | **405.7137** | **36** | **3.11E-67** | **4.87658** |  |
| ***FGFR3*** | **18.10387** | **5.327181** | **9.46E-52** | **275.6075** | **30** | **6.02E-49** | **12.77669** |  |
| ***MAF*** | **15.65282** | **8.560767** | **2.37E-29** | **139.9843** | **20** | **9.59E-27** | **7.092049** |  |
| ***FAM30A*** | **9.045972** | **11.92949** | **0.000101** | **15.3173** | **137** | **0.006506** | **-2.88352** | **lncRNA** |
| ***SCARNA22*** | **6.303514** | **1.248115** | **8.26E-55** | **295.9046** | **36** | **6.13E-52** | **5.055398** | **snoRNA** |
| ***TMEM121*** | **4.976042** | **2.771555** | **0.000712** | **11.56931** | **25** | **0.031084** | **2.204486** | **No results NetworkAnalyst** |
| ***CRIP1*** | **10.76942** | **8.123555** | **0.000255** | **13.52904** | **20** | **0.013336** | **2.645864** | **No results NetworkAnalyst** |
| ***CCND2*** | **18.39943** | **8.442989** | **1.48E-06** | **23.61886** | **5** | **0.00015** | **9.95644** | **Nothing Sig** |
| ***USP49*** | **16.51937** | **9.494438** | **3.81E-35** | **172.9638** | **5** | **1.70E-32** | **7.024937** | **No results NetworkAnalyst** |
| ***CCND3*** | **15.75047** | **11.35996** | **1.33E-11** | **47.47981** | **5** | **2.37E-09** | **4.390507** | **Nothing Sig** |

# **Supplementary Figure 1: linear regression between SV and leucocyte telomere length (A) and tumor telomere length (B)** showing a correlation between SV and LTL and TTL

# **Supplementary Figure 2: Loess regression of telomere versus number of structural events:** showing that short telomeres are correlated with a high number of SV

# **Supplementary Figure 3. Correlation between percentage of APOBEC signature and number of structural variants:** showing a small but significant correlation between SVs and percentage of APOBEC mutational signature.

# **Supplementary Figure 4. Distribution of the number of SV per patients:** suggesting a tail of patients representing 10% of the total have high SV load.

# **Supplementary Figure 5. Relationship of structural and copy number events** showing a significant correlation between canonical translocations, recurrent copy number aberrations, mutations and complex rearrangements using different levels of significance. A. Bayes Factor ≥1, B. Bayes Factor ≥10, C. Bayes Factor ≥100, and D. Bayes Factor ≥1000. These data suggest complex rearrangements are associated with DNA instability markers.

# **Supplementary Figure 6. Pie chart of the proportion of patients with *RB1, TP53* biallelic inactivation and telomere attrition (<4.100kB) by varying structural event groups** A. High structural load. B. Chromothripsis, C. Chromoplexy, D. Templated insertion. sTTL: short Tumor telomere length: showing that chromothripsis, chromoplexy high structural loads are associated with DNA instability whereas templated insertion is not.

# **Supplementary Figure 7. Comparison of the number of SVs at presentation (blue) and relapse (red):** suggesting there are more SVs at relapse**.**

# **Supplementary Figure 8. Complex SV detection over time.** Four time points are shown for the same patient (A-D and E-F). Although the complex t(2; 3; 12; X) SV was only detected at time point 4 (D and H) by Manta, manual investigation identified reads supporting the presence of the event at all time points (A-E: first, B-F: second, C-G: third). This could be explained by its presence and sub-clonal expansion rather than it being a new event at T4.


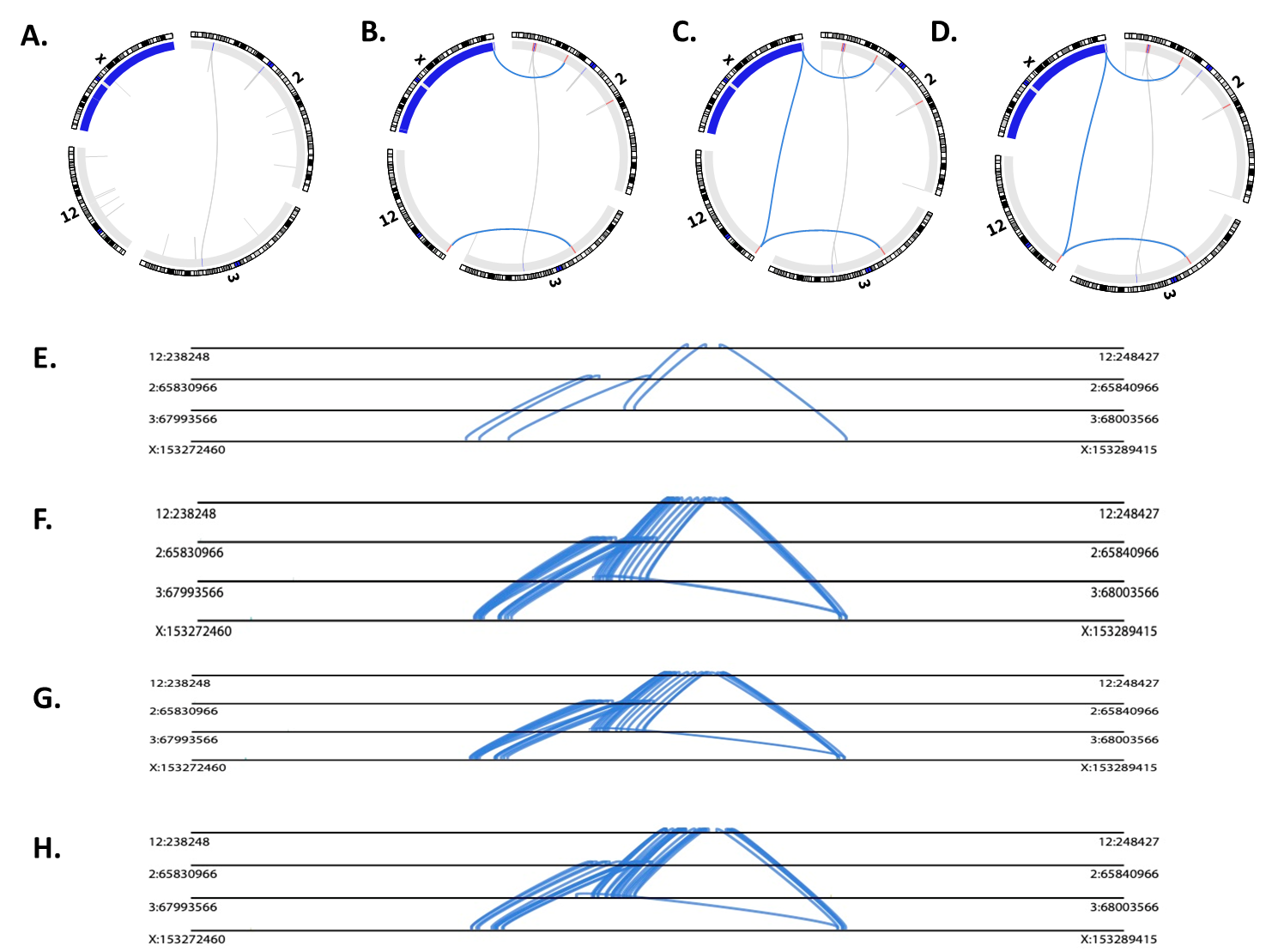


# **Supplementary Figure 9. Expression changes and between cases with TAD-TAD rearrangements and those that have none using U266 defined TADs. The x-axis scale is the inverse log10 p-value using a log-ed scale and the y-axis scale is the log2 fold change, A. Overall B. In detail view of the center region.**


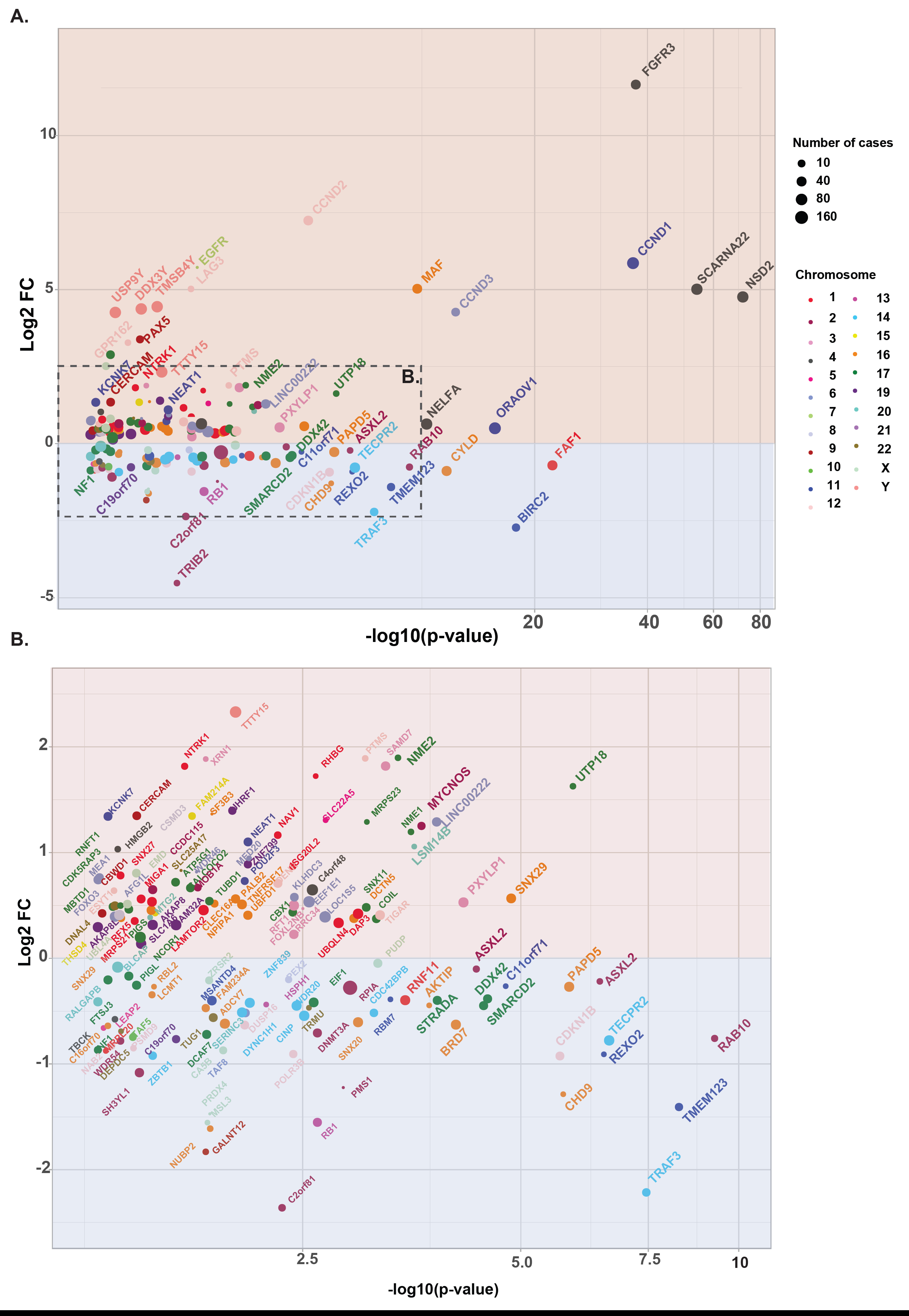


# **Supplementary Figure 10. Genes deregulated by the t(4;14) and their fold change, n= number of cases.**


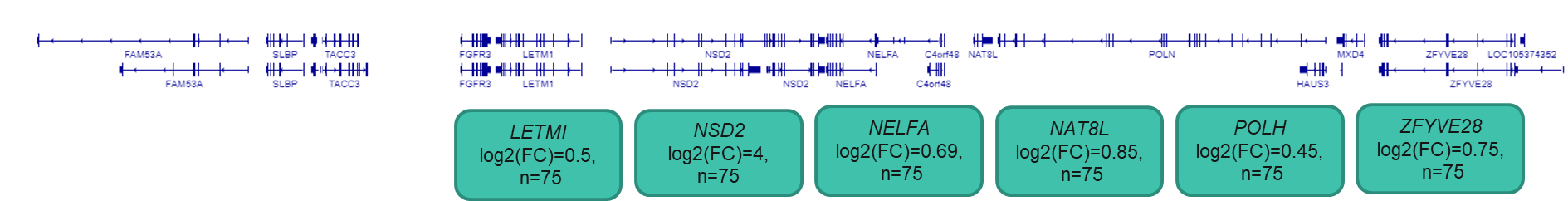


# **Supplemental Figure 11: Expression of MAP3K14 according to the localization of the individual breakpoint:** Showing that the level of expression is dependent upon the site of the breakpoint in relation to the gene locus. A. Map depicting the relationship between the gene and related TADs. B. Ranked MAPK14 expression. In green samples with a breakpoint located in the 5’ TAD; in red with those with a breakpoint located in the red TAD suggesting.


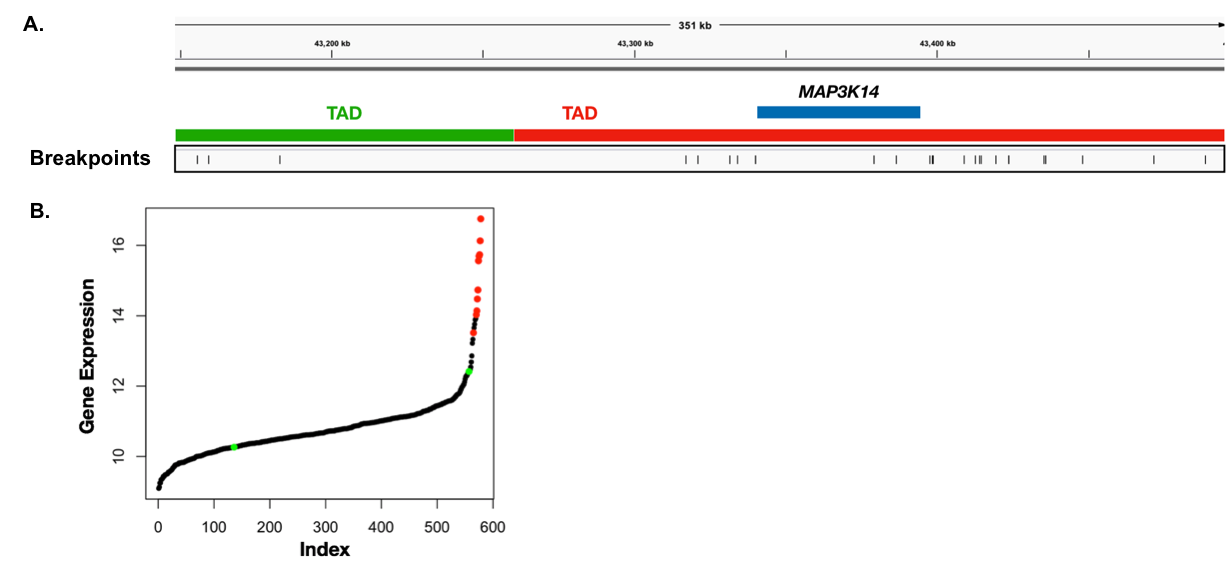


# **Supplemental Figure 12: Kaplan Meier survival curve showing the impact of a high structural load on PFS (A) and OS (B).**
